# A cohort follow-up study for diabetic retinopathy screening incidence in the North Denmark Region

**DOI:** 10.1007/s00592-023-02146-4

**Published:** 2023-07-12

**Authors:** Tobias P H Nissen, Peter Vestergaard, Henrik Vorum, Christian Torp-Pedersen, Kristian Aasbjerg

**Affiliations:** 1 Department of Ophthalmology, Aalborg University Hospital, Hobrovej 18-22, 9000 Aalborg, Denmark; 2 Steno Diabetes Center North Denmark, Hobrovej 18-22, 9000 Aalborg, Denmark; 3 Department of Endocrinology, Aalborg University Hospital, Hobrovej 18-22, 9000 Aalborg, Denmark; 4 Department of Cardiology, Nordsjællands University Hospital, Hillerød, Denmark; 5 Department of Public Health, University of Copenhagen, Copenhagen, Denmark; 6 Himmerland Eye Clinic, Aalborg, North Denmark Region Denmark

**Keywords:** Screening incidence, Diabetic retinopathy, Diabetes

## Abstract

**Aims:**

To evaluate diabetic retinopathy (DR) screening incidence in a universal healthcare system.

**Methods:**

Registry-based cohort study based on a Danish regional population from 2009 to 2018. Individuals with diabetes were identified by medication. Screening attendance was estimated by surrogate measures using local and nationwide databases reported by cumulative incidence.

**Results:**

18,832 patients were included. By the end of the first year, the cumulative incidence of screening for DR was 60.2% and by the end of the second year 74.2%. The cumulative incidence was 93.9% overall, 97.7% for patients with type 1 diabetes (T1D) and 93.4% for patients with type 2 diabetes. Screening proportions per 1, 2 and 5 years were calculated. Females, patients with T1D, and patients attending screening at hospitals had a higher Hazard Ratio of 1.084, 1.157, and 1.573, respectively. The Cochran–Armitage trend test indicated increased screening frequency from 2009 to 2018. Validation of DR screening was done at hospitals with a mean positive predictive value of 86.78%. Cumulative incidence curves showed a small right shift when censoring the first, second and third screening visits.

**Conclusions:**

Nearly all patients were screened for DR over a 5-year timespan. Female patients with T1D who attended screening at hospitals were significantly more likely to be screened. Validation of screening visits at hospitals was reported with a high mean positive predictive value. Most other studies, to the best of our knowledge, only report screening attendance for patients already enrolled in a DR screening programme. This study describes the overall screening attendance for the total eligible diabetes population.

## Introduction

The prevalence of diabetes is increasing worldwide [1], and consequently the prevalence of diabetic retinopathy (DR) is also rising [2]. Patients with diabetes should thus have regular checks for DR so that early signs can be identified before the patient experiences any visual disturbances. Early detection and treatment of DR are important as they ensure the prevention of permanent damage occurring in the retina [3]. The global prevalence of DR is just above 20% according to a systematic review from 2021 [4]. In 2019, the worldwide diabetic population worldwide was 537 million adults. This number is expected to rise to 783 million by 2045 according to the International Diabetes Federation [1]. In 2019, the actual number of people with DR was 119 million. If the prevalence of DR remains the same, this number will be around 174 million in 2045 [4, 5].

Screening for DR is a simple procedure involving standard ophthalmological examination techniques such as fundus photo, ophthalmoscopy and/or optical coherence tomography. Signs of retinopathy, such as small dot haemorrhages, microaneurysms, hard exudates, cotton wool spots and neovascularisations are only detected via screening, and patients rarely experience a change in visual acuity. Only severe late-stage DR, such as macula oedema, bleeding due to neovascularization or retinal detachment due to fibrous proliferation impair visual acuity to a degree that the patient will notice, and early stages can therefore only be detected via screening. Despite the simplicity of screening and the benefits of early detection, it is well known that the rate of patients being screened for DR is not very high. The American Academy of Ophthalmology (AAO) reports that only around 60% of patients with diabetes come for screening [6]. Patients who do not adhere to screening recommendations are at high risk of possibly ending up with permanent visual impairment due to severe and proliferative DR [7].

This paper aims to estimate screening attendance by cumulative incidence over a 10-year period (i.e., 2009–2018) for patients who attended a free national DR-screening programme at hospitals and/or at private ophthalmologists in the North Denmark Region. To the best of our knowledge, a study such as this has not been done before. From 2010 to 2017, patients in Denmark were generally given up to only 2-year screening intervals depending on their retinopathy status [8]. Through the utilisation of Danish National Health Registries [9, 10], we identified individuals with diabetes (T1D and T2D) in the Danish National Screening programme for DR and validated the models for detecting screening based on general nationwide registries when compared to a local high-quality DR screening database. We also investigated the likelihood for screening with reference to sex, diabetes type and screening location.

## Methods

### Study design and population

In Denmark, every resident is assigned a unique personal civil registration number at birth which is linked across nationwide registries at an individual level. [10–12]. The healthcare system is free for all residents, and all service providers are required to report their services to the appropriate registries for reimbursement from the government thus incentivizing and ensuring high-quality data. Diabetes screening is performed at hospitals (primarily patients with T1D) and private clinics (primarily patients with T2D).

### Population

The study was designed as a cohort study from 2009 to 2018. The North Denmark Region was chosen due to the presence of a high-quality database that addressed screening for DR which covered the region’s screened diabetes population.

### Registries used

The Danish National Prescription Registry [13, 14] holds all information about the type of dispensed medicine, date that medicine is dispensed and number of prescriptions.

The Danish National Health Service Registry [15] contains records of all services performed in the private branch of Danish healthcare that are reimbursed by the government. The number of ophthalmologists not working in connection to the public system in Denmark is extremely low because individuals must pay for an otherwise free service.

The information about out-patient clinics can be found in The Danish National Patient Register which holds all information regarding hospitals visited, departments visited, length of stay, dates of stay, type of visit, diagnosis and procedures performed [10].

The North Denmark Region has a local high-quality DR screening database that contains fundus photos that have date stamps. This database was used for comparing and validating DR screening at hospitals.

### Identifying people with diabetes

To identify the Danish diabetes population, the Anatomical Therapeutic Chemical Classification System (ATC) codes [16] for diabetes were used. For anti-diabetics with insulin and insulin analogues, MA10A was used. For anti-diabetics without insulin, MA10B was used. Some patients with T2D were also prescribed insulin or analogues but nearly all patients with T2D had at some point been prescribed a non-insulin. It is not recommended to prescribe hypoglycaemic agents for T1D according to official Danish guidelines [17].

T1D was defined as patients with redeemed prescriptions for insulin or insulin analogues, and those never having redeemed any prescription for non-insulins.

T2D was defined as prescriptions for non-insulin medicine and insulin.

Each patient had to have a minimum of two dispensed prescriptions within 180 days in order to be defined as having diabetes. The second prescription had to be dispensed in 2009 and was used as the inclusion date.

We filtered and discarded women under the age of 40 who only received Metformin (ATC-code A10BA02) to filter out patients with polycystic ovarian syndrome (PCOS) or endometriosis [18, 19].

### Screening locations

By default, patients with T2D were screened at private ophthalmologists, and patients with T1D are primarily screened at hospitals. Patients with T2D were only eligible to be screened at hospitals if they were referred by a private ophthalmologist.

### Identifying screening in a hospital setting

We defined screening performed at the hospitals as those registered with one ICD-10 code (UCXA). Only patients with diabetes and non-acute contacts were included from hospitals in the North Denmark Region.

### Hospital screening-local high-quality DR screening database

At the beginning of 2000, a local high-quality DR screening database created to keep track of patients who require screening for DR was established in the North Denmark Region. The database consists of fundus photos, DR grades, biochemistry, date of visit and visual acuity. The database was linked to other registries by using the patient’s civil registration number to compare the above-mentioned population in hospitals. The date of visit was extracted and imported to Statistics Denmark [20] to merge with the above-mentioned registries (4521 patients from the defined diabetes population were in the database). Validation of the screening at hospitals was done by comparing yearly visits in the database to the yearly visits identified through The Danish National Patient Register. This was done due to uncertainty of the definition of patients screened at hospitals in the registers.

### Private ophthalmologist

Since 2015, individuals screened by private ophthalmologists have been registered with a specific service code (190,112). As data in the registries are still sparse, an indirect estimation of screening by private ophthalmologists was done.

The definition of screening for DR by private ophthalmologists required a visit to a private ophthalmologist and a diabetes diagnosis as defined above (14,470 patients had a visit at a private ophthalmologist during this period).

### Identifying screening at both hospital and private ophthalmologists, and patients with no screening

All datasets were merged to combine the information. Diabetes, sex, resident status, birth, death and screening per year were reviewed by one of the three sources of screening. Patients who moved out of the North Denmark Region during the period were censored, and the date of censoring was chosen as 30 June in the year of the move, as no data for the date of the move was provided. Patients who died were censored on the day of death. For calculating the overall cumulative incidence, the first date of screening was selected whether it was from the local database, hospital or private ophthalmologist. When cumulative incidence for a single screening location was calculated, the first date of screening recorded at the respective location was used.

### Data analysis

Data management was conducted using the SAS Statistical Software package for Windows, version 9.4 (SAS Institute, Cary, NC, USA). Proc lifetest and the cumulative incidence function were also used. Gray’s test was used to test the difference in the cumulative incidence curve (CIC) in multiple groups. The PHREG function was used for the cause-specific hazard ratios (HR) [21] and for calculating both the Chi^2^ and Cochran–Armitage estimate. For bar plot, the Exact Binominal function (Clopper–Pearson) was used. Gray’s Test for Equality calculated the cumulative incidence curves.

## Results

### Total population

From 1 January 2009 to 31 December 2018, a total of 580,515 individuals lived in the North Denmark region. Of these, 18,832 individuals (43.9% female) were included, as they redeemed at least two prescriptions related to diabetes with their second prescription being in 2009. Of the 18,832 individuals, 2627 (13.9%) were diagnosed with T1D (Table 1), and 16,205 individuals (86.1%) were diagnosed as having T2D.

**Table 1:** Epidemiological characteristics of the overall included population

Baseline 2009		Sex		Total
		Female	Male	
T1D	Participants	1112	1515	2627
	Percent	13.43%	14.36%	13.95%
	Median age (IQR)	46.15 (30.64)	45.16 (29.02)	45.67 (29.74)
T2D	Participants	7168	9037	16,205
	Percent	86.57%	85.64%	86.05%
	Median age (IQR)	67.77 (18.36)	64.72 (16.41)	65.91 (17.33)
Total	Participants	8280	10,552	18,832
Age at death				
T1D	Median (IQR)	74.4 (22)	69.8 (25.3)	71.62 (23.3)
T2D	Median (IQR)	82.99 (13.35)	77.99 (14.38)	80.19 (14.5)

Interquartile range (IQR) Q1–Q3

Kruskal–Wallis test for significant difference in medians between age at death of T1D versus T2D: *p* < 0.0001

The median age differs in T1D and T2D and is reported in Table 1 along with the Interquartile range Q1–Q3 (IQR).

### Cumulative screening incidence

From 1 January 2009 to 31 December 2018, a total of 18,832 participants were eligible for screening. The mean age of death and IQR for T1D and T2D are reported in Table 1.

**Fig. 1:**
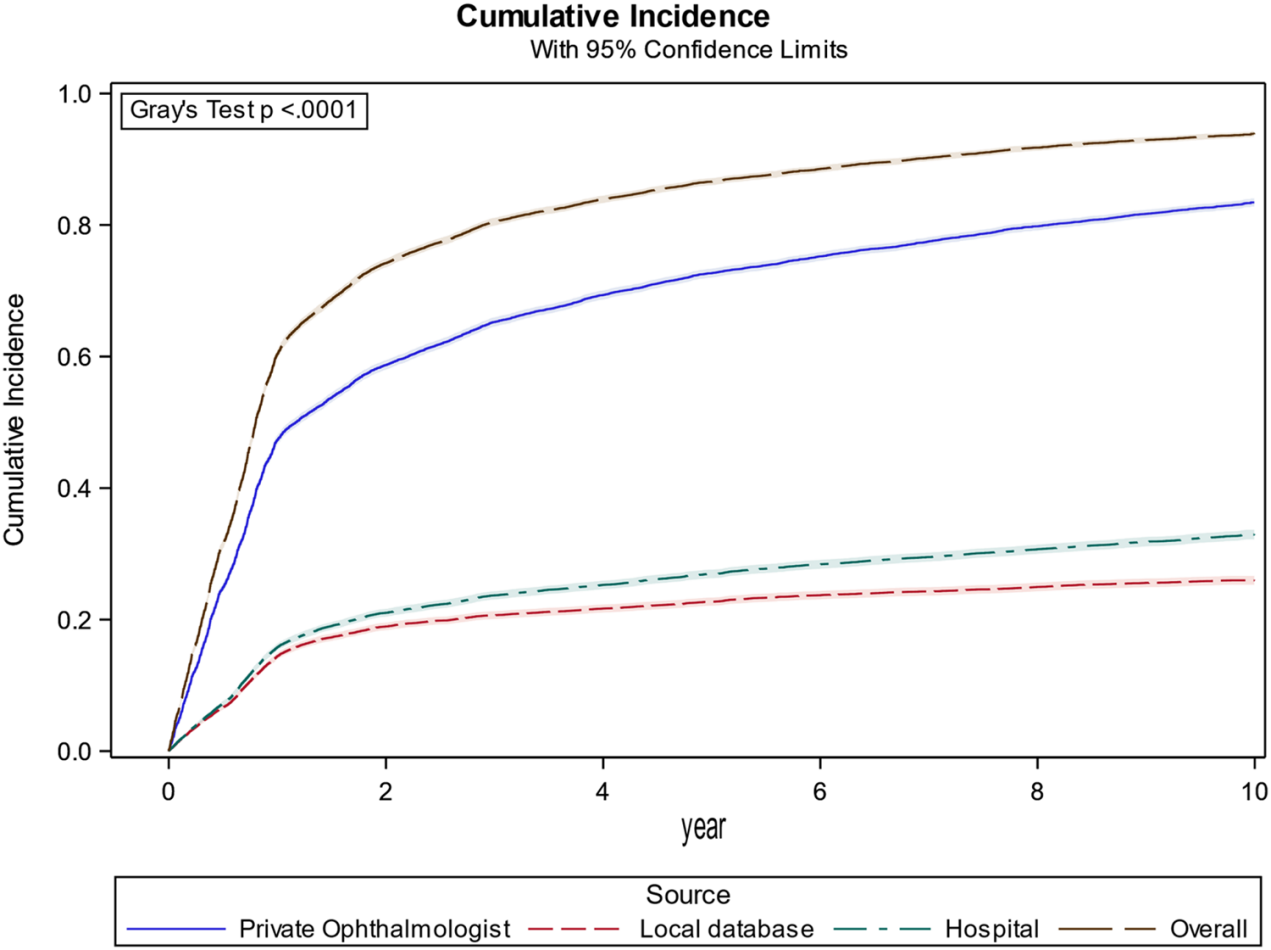
X-axis: years from 1 Jan 2009 and onward. Y-axis: cumulative Incidence of patients who had seen an ophthalmologist. ‘Overall’ is the overall cumulative incidence for all data sources. ‘Hospital’ is the cumulative visits at a hospital. ‘Private Ophthalmologist’ is cumulative visits with a private ophthalmologist. ‘Local database’ is the cumulative known screening visits at a hospital

By the end of the first year, the cumulative incidence was 60.4% (95% CI 59.8–61.1); and by the end of the second year, the cumulative incidence rose to 74.2% (95% CI 73.6–74.8). At the end of the ninth year, the cumulative incidence was at 93.9% (95% CI 93.4–94.3), which expresses the fraction of the eligible population that had seen an ophthalmologist (Fig. 1, ‘Overall’) and thereby were defined as having *been screened for DR.*

### Strata by sex and diabetes type

**Fig. 2:**
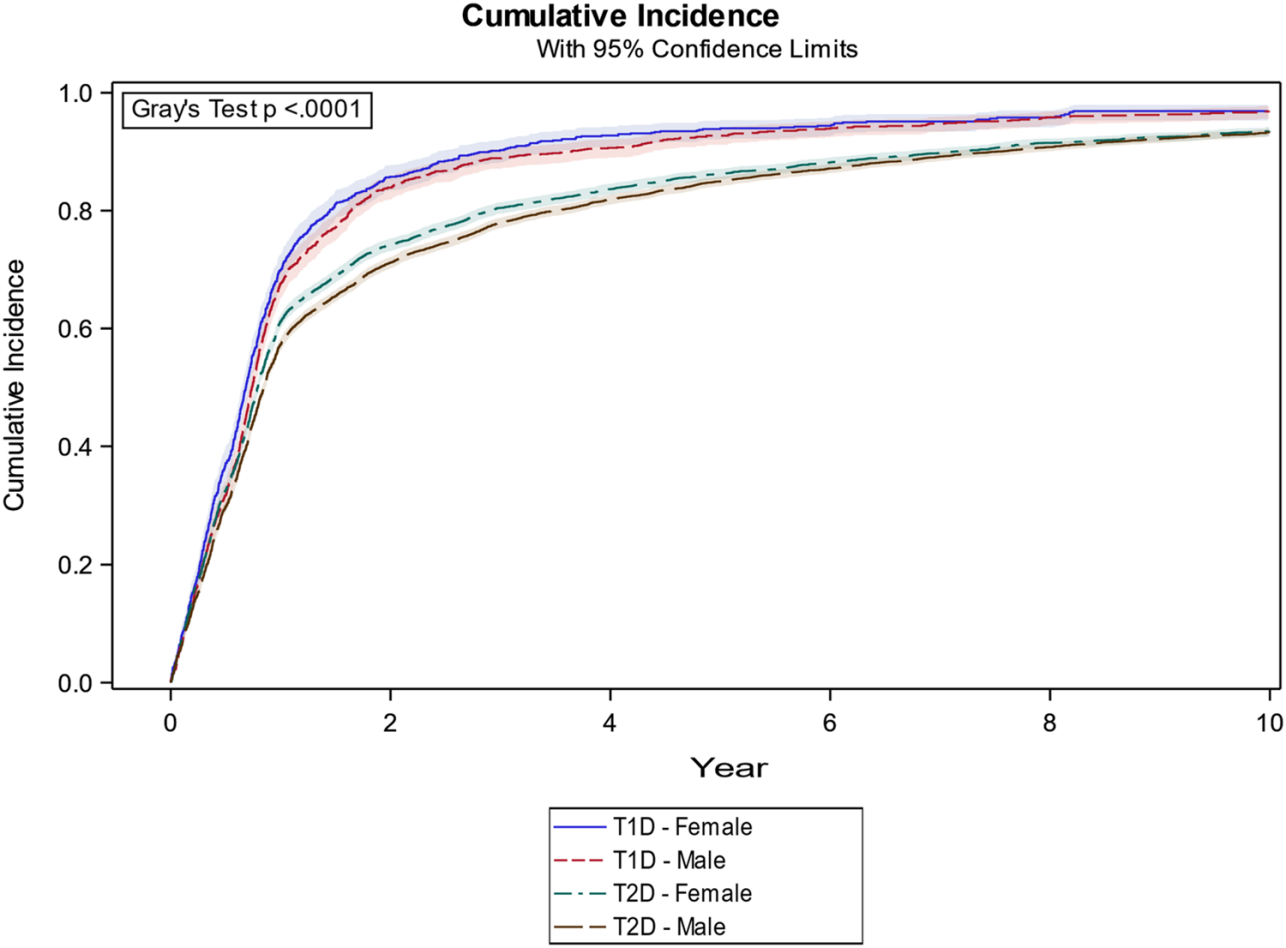
X-axis: years from 1 Jan 2009 and forth. Y-axis: cumulative Incidence of patients who had seen an ophthalmologist based on all data sources

Direct readings from the cumulative incidence function estimates with strata on sex and diabetes for the first and second year and for diabetes for the fourth and ninth year (Fig. 2).

At the end of the first year, 70.0% (95% CI 67.1–72.6) of females and 67.7% (95% CI 65.2–70.0) of males with T1D versus 61.2% (95% CI 60.0–62.4) of females and 57.3% (95% CI 56.3–58.3) of males with T2D had been screened.

At the end of the second year, 85.8% (95% CI 83.5–87.7) of females, 83.9% (95% CI 81.9–85.7) of males with T1D versus 74.2% (95% CI 73.1–75.2) of females, 71.1% (95% CI 70.2–72.1) of males with T2D had been screened.

At the end of the fourth year, the CIC (both females and males) showed 91.5% (95% CI 90.3–92.5) of patients with T1D and 82.7% (95% CI 82.0–83.2) of T2D patients had been screened.

At the end follow-up and the ninth year, (both females and males) 96.8% (95% CI 96.0–97.6) of patients with T1D and 93.4% (95% CI 92.8–93.9) of patients with T2D had been screened.

Females (T1D and T2D) had an HR (Table 2) of 1.084 (95% CI 1.051–1.119) which means that females in general were more likely to be screened than males. HR for screening at hospitals for both T1D and T2D was 1.157 (95% CI 1.100–1.217), which translates to patients who were screened at hospitals being more likely to be screened in general than those who went to a private ophthalmologist.

**Table 2:** Hazard ratio (HR) on covariates was calculated for the incidence curve (Fig. 1 and Fig. 2) to estimate the covariate effect on DR screening attendance

Variable	Inclined towards	Wald chi^2^:* p*	HR	HR 95% CI	
Diabetes	T2D < T1D	< .0001	1.157	1.100	1.217
Sex (T1D and T2D)	Male < Female	< .0001	1.084	1.051	1.119
Sex (T1D)	Male < Female	0.03	1.093	1.008	1.186
Sex (T2D)	Male < Female	< .0001	1.087	1.051	1.124
Age/decade (T1D, T2D)	Increasing age	< .0001	1.023	1.012	1.035
Age/decade (T1D)	Increasing age	< .0001	1.074	1.052	1.096
Age/decade (T2D)	Increasing age	0.25	1.007	0.995	1.020
DR location (T1D, T2D)	P.O. < Hospital	< .0001	1.573	1.510	1.639
DR location (T1D)	P.O. < Hospital	< 0.001	1.642	1.504	1.792
DR location (T2D)	P.O. < Hospital	< .0001	1.560	1.490	1.634

For the variable ‘Diabetes’, more patients with T1D than T2D were screened

*CI* confidence interval, *P.O* private ophthalmologists

### Strata by data source and diabetes

**Fig. 3:**
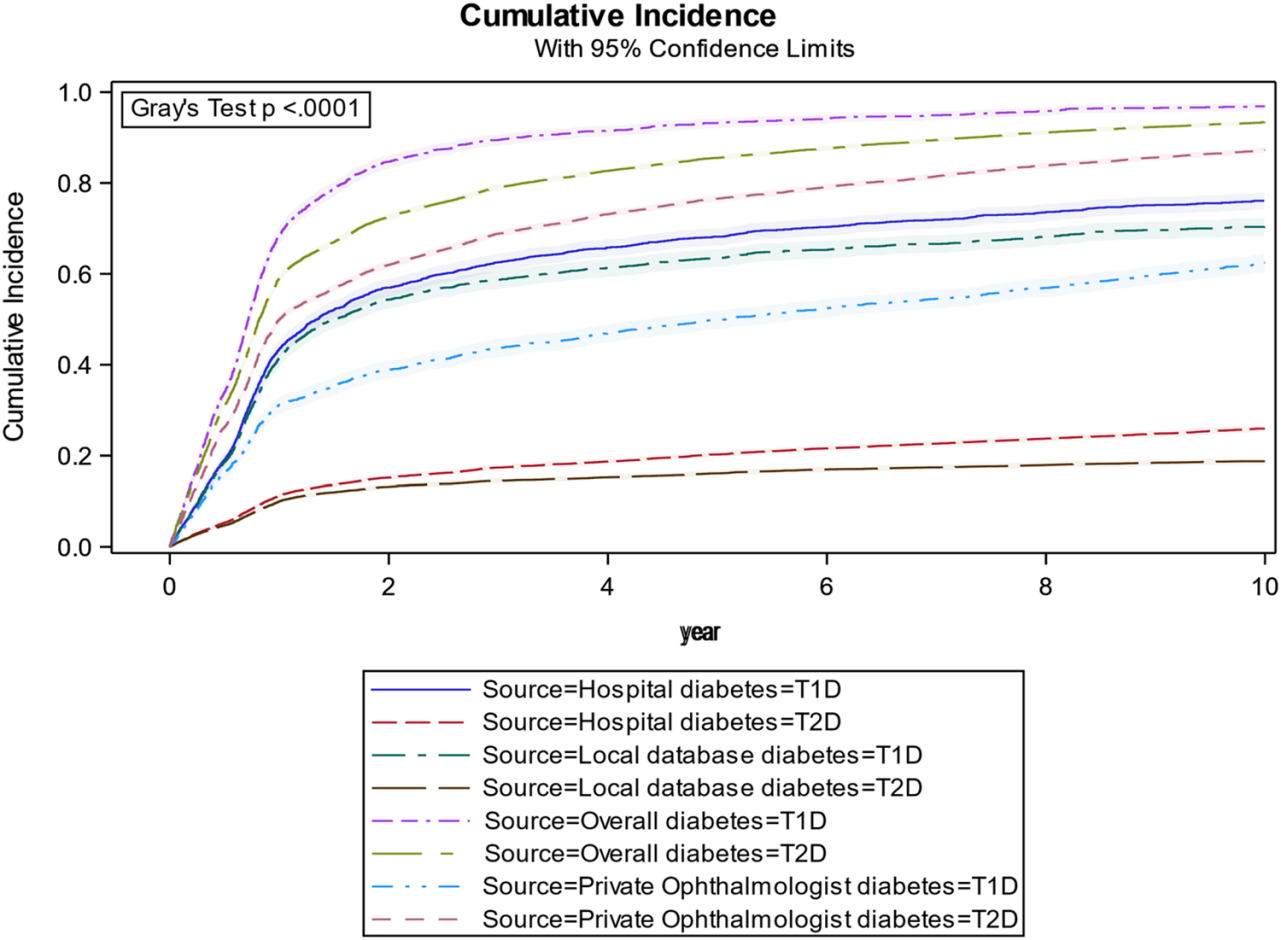
X-axis: years from 1 Jan 2009 and forth. Y-axis: cumulative Incidence of patients who have seen an ophthalmologist stratified on the respective data sources. ‘Overall’ is the overall cumulative incidence for all data sources. ‘Hospital’ is the cumulative visits at a hospital. ‘Private ophthalmologist’ is the cumulative number of visits with a private ophthalmologist. ‘Local database’ is the cumulative known screening visits at a hospital. All data sources are stratified based on diabetes type

When stratifying by data source and diabetes (Fig. 3), few patients with T2D were seen at the hospital (T2D-Local database, T2D-Hospital) with a total cumulative incidence of 18.7% (95% CI 18.1–19.4) and 25.9% (95% CI 25.2–26.7), respectively. At the end of the study, 62.4% (95% CI 60.4–64.4) of the patients with T1D had visited a private ophthalmologist.

### Screening incidence per year

**Fig. 4:**
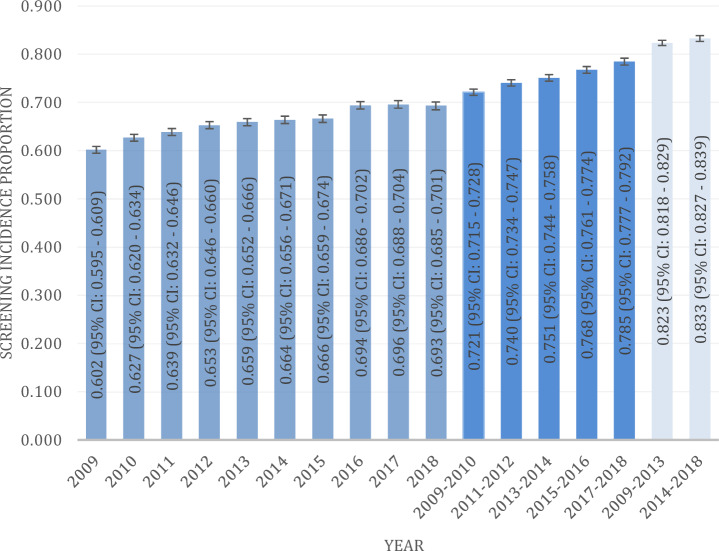
Cumulative screening incidence proportion of the screened versus non-screened population. The proportion of the screened population is shown by 1-year intervals (medium blue), 2-year intervals (dark blue) and 5-year intervals (light blue). 95% confidence interval (CI) (color figure online)

The incidence proportion of the eligible diabetes population rose from 60.2% (95% CI 59.5–60.9) per year (2009: 11,330/18,832) to 79.3% (95% CI 68.5–70.1) per year (2018: 8,653/12,489) over the 10-year time span (Fig. 4). The Likelihood Ratio Chi^2^ was 606.4, *p* < 0.0001 with nine degrees of freedom. The Cochran–Armitage Trend Test showed *Z* − 23.8, and *One-sided p* < *Z* 0.0001 which indicates a statistically significant positive DR screening trend from 2009 to 2018.

### Validation of methodology for finding DR screenings at hospitals

The mean positive predictive value (PPV) was calculated for the diagnosis of DR screening at hospitals versus ground truth, which was the local database, for the years 2009–2018. PPV: 86.78% (95% CI 86.76–86.81).

## Discussion

This large registry-based regional cohort-based study, which included more than 18,800 patients with diabetes, found a high cumulative DR screening incidence and increasing trend over 10 years with up to 78% attendance (2017–2018, Fig. 4) by a two-year interval (as recommended by national guidelines).

We found an increase in screening attendance by year throughout the study (Table 2). Furthermore, we demonstrated that nearly all patients with diabetes see an ophthalmologist and more than 60% of patients with T1D are at some point seen by a private ophthalmologist. This is being done even though DR screening of patients with T1D is mainly performed at hospitals in Denmark.

Only about 74% of patients had seen an ophthalmologist two years after the study start, with a general discrepancy between patients with T1D and T2D being observed throughout the study. Screening guidelines for DR are outlined in guidelines from the International Council of Ophthalmology [5], but guidelines are not necessarily followed as shown above and across multiple studies in a Cochrane review [22]. This may be due to individualised DR screening intervals or lack of patient compliance.

When adjusting for T1D and T2D, those who are diagnosed with T1D have a higher and steeper incidence curve (Fig. 2). Conversely, the curve for patients with T2D seems to flatten out after year one. We are not aware of other studies describing this phenomenon. Patients with T1D and females, in general, attended DR screening more often than patients with T2D and males in general. This was confirmed by the HR (Table 2) where females were significantly more likely to attend screening with an HR of 1.084 (95% CI 1.051–1.124). The group with T1D also showed a significantly higher HR of 1.157 (95% CI 1.100–1.217) compared to patients with T2D. Here it can be speculated that these patients in general with T1D have higher disease awareness than those patients with T2D. As reported by AAO in the introduction, only about 60% of patients with diabetes attend DR screening [6]. However, such data can vary from country to country [23, 24] due to the low-grade quality of evidence which is the result of the inconsistency reported. A Cochrane review from 2018 [22] with 329,164 participants (mainly from USA and Europe) reports 47.2% attendance with usual care and 58.0% attendance with intervention. The National Health Service in England reported 82.4% attendance from 2016 to 2017 [25]; however, it is unclear whether attendance was by the total diabetes population or just by the enrolled population. Our study, on the other hand, includes the whole diabetes population and not just the population already enrolled in a screening programme.

**Fig. 5:**
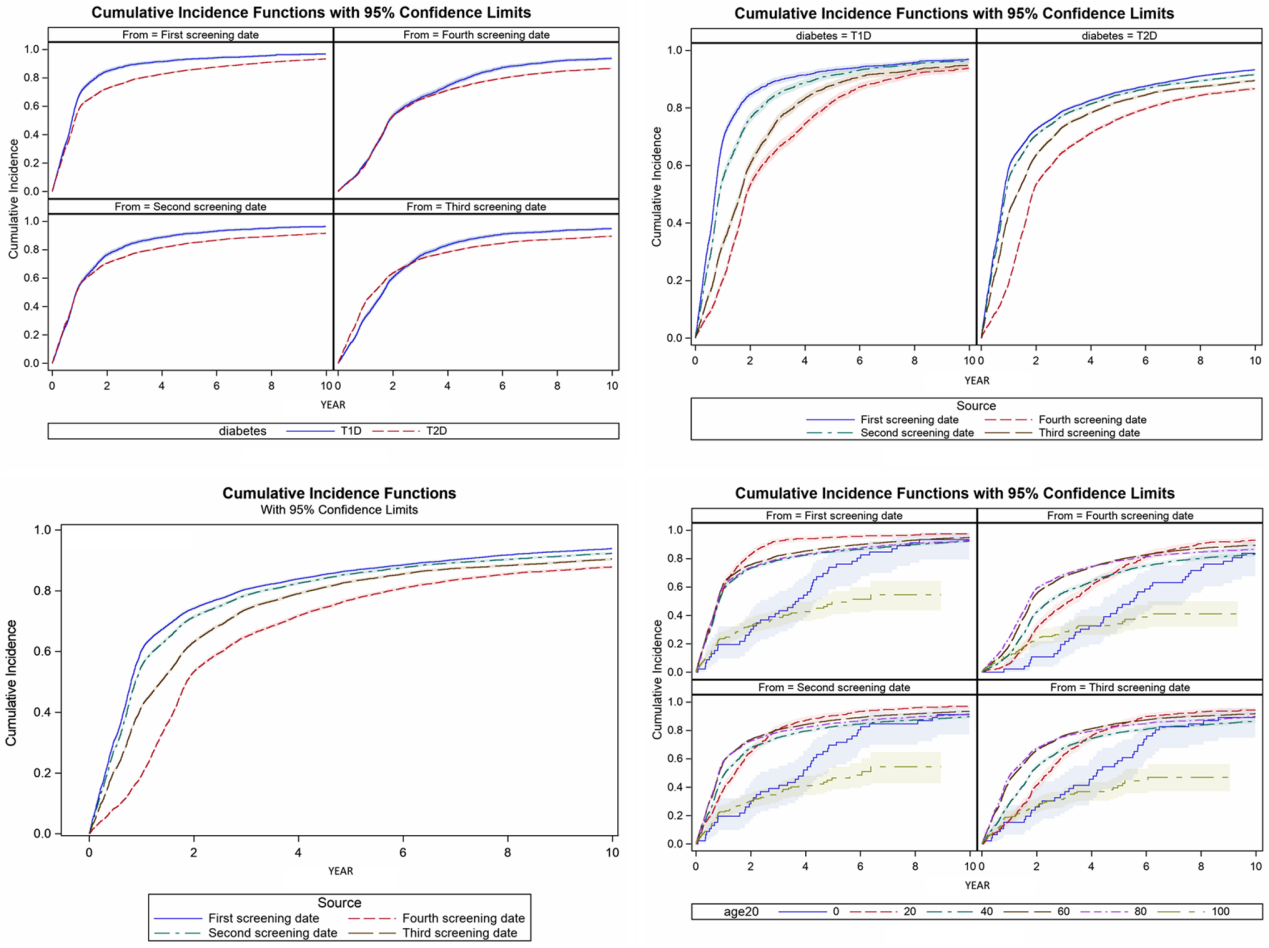
Cumulative incidences curves for screening, where the start date is varied from the first to the fourth screening date, whereby censoring up to the first three screening dates. Upper left: stratified on diabetes type and screening date. Upper right: screening date and diabetes type. Lower left: screening date and combined diabetes type. Lower right: age in 20 years intervals (age 20 = 20 years intervals) and screening date. Stratifying screening date (censoring first, second and third screening) shows a minor decline in the overall cumulative incidence. This indicates the cumulative incidence curve is not driven by only a few individuals. Age 0 (solid blue) containing individuals between age 0 and 19, and age 100 (stippled yellow), containing individuals between age 100–119, do have fewer screening visit’s. Age20 = 20 year interval with starting age marked (color figure online)

Age by decade is not deemed a strong estimator of screening attendance in this study but is described as important in other studies [26, 27]. When HR for sex was stratified in T1D and T2D, there was still a slightly higher HR ratio for females/males with T1D than females/males with T2D who attended DR screening. In Fig. 5, we noticed the cumulative incidence rise towards a high endpoint at year 10 when varying from the first to the fourth available screening date. The general tendencies described in the discussion and results seem to be confirmed even when the first screening date is varied by censoring (Fig. 5).

The location of the screening appears to influence screening attendance, regardless of whether patients had T1D or T2D. Both groups were significantly more likely to be screened if they were screened at hospitals, which to our knowledge has not been described elsewhere. This may indicate that patients who attend DR screening at hospitals get better patient education, are more informed or are followed up with on more than patients who attend DR screening at private ophthalmologists.

Patient disease awareness could be insufficient as reported by a study from Hong Kong [28]. Several studies [29, 30] focus on reasons for non-attendance (dropouts and never attendance). An overview of the total screening attendance of the Danish diabetes population does not exist, as The Danish Registry of Diabetic Retinopathy only reports patients who have been screened and not the total diabetes population [31]. Additionally, this is the first study describing cumulative screening incidence and proportion for a regional population in Denmark.

### Strength and limitations

The strength of this study is the large number of patients, the availability of data from private ophthalmologists and the possibility to have a local database matched by civil registration number in order to get a more accurate estimation of patients who are screened at hospitals. Furthermore, the region is well-covered with screening sites and ophthalmologists. A high PPV for screening at hospitals was found when compared to the local database and could be used for further register studies on a nationwide level. The general trend was confirmed even when varying the first screening date, which implies that the results were not driven by a few individuals.

The limits of the study are the general limitations of using registries like the ones defining the diabetes population where it is known from the clinic that some patients redeem their prescriptions for large amounts of medicine for more than 180 days of use. Patients who do not redeem prescriptions for medication are not included.

A main challenge is how DR screening is defined. We cannot be sure that a visit to an ophthalmologist or hospital effectuates a DR screening. The validation of DR at hospitals may not be generalized at a national level due to the possibility of different coding practices. There is a slight over reporting regarding the way DR screening is defined at hospitals which can probably be explained by false positives due to the methodology.

### Conclusion

This study of more than 18,800 patients in Denmark found an overall high DR screening attendance in the diabetic population including never attendants with a statistically significant increasing incidence trend. We found it important to report on patients who have never attended screening, as this might be a less highlighted subject in the literature. T1D patients, patients who attend screening at hospitals and female patients were statistically significantly more likely to be screened for DR. Males with T2D screened at private ophthalmologists were less likely to be screened. The validation of the method to find yearly screening visits at hospitals showed a high mean PPV but should be cautiously used in other regions in Denmark as there is a possibility of different coding practices. Censoring up to the first three screening dates (Fig. 5) did not change the general tendencies of the study.
